# The Accuracy and Reliability of the Photometric Method—A New Noninvasive Tool for Assessing Frontal Lower Limb Alignment

**DOI:** 10.3390/jcm14124244

**Published:** 2025-06-14

**Authors:** Anna Fryzowicz, Jan Szymczak, Paweł Koczewski

**Affiliations:** 1 Department of Biomechanics, Poznan University of Physical Education, ul. Królowej Jadwigi 27/39, 61-871 Poznań, Poland; 2 Pediatric Orthopedic and Traumatology Department, Dega Hospital in Poznań, Poznan University of Medical Sciences, ul. 28 Czerwca 1956 r. 135/147, 61-545 Poznań, Poland

**Keywords:** knee osteoarthritis, varus knee, valgus knee, noninvasive, photography

## Abstract

**Background/Objectives:** The aim of this study was to establish the reliability and accuracy of a new noninvasive tool for FLLA (frontal plane lower limb alignment) assessment: a photometric method. **Methods**: Sixty-seven subjects (31 males, 36 females, age 11–47 years) participated in the study. Seventeen subjects with orthopedic disorders were marked with radiopaque markers over the anterior superior iliac spines and femoral condyles. One pelvis-to-floor radiograph and one photograph were taken in the same standardized standing position. The hip–knee–ankle (HKA) angle (radiography) and the pelvis–knee–ankle (PKA) angle (photography) were measured by one rater. In 50 healthy participants, anterior superior iliac spines and femoral condyles were marked, and two pelvis-to-floor photographs were taken in a standardized standing position. The PKA angle was measured two times by three raters. The accuracy of the photometric method was tested with Pearson’s correlation coefficient, simple linear regression, and Bland–Altman analysis. The reliability was tested with ICC(2,k) and Bland–Altman analysis. **Results**: The HKA angle was on average 3.9° more varus than the PKA angle, with a high correlation between measures (r = 0.97, *p* < 0.0001) and limits of agreement between −1.300 and −6.482. Intrarater (ICC(2,k) > 0.972), interrater (ICC(2,k) = 0.991), and test–retest (ICC(2,k) = 0.980) reliability were excellent. **Conclusions**: The photometric method is promising as a reliable and accurate noninvasive tool for assessing FLLA. Its accuracy across different study groups has yet to be confirmed in a larger cohort. The advantage of the presented photometric method is the use of the easily palpable anterior superior iliac spine as the proximal femoral axis point.

## 1. Introduction

Frontal plane lower limb alignment (FLLA) deformity is associated with a higher risk of the development [1,2] and progression [1,3] of knee osteoarthritis, which causes pain and disability [4,5]. For FLLA assessment, weight-bearing full-length lower limb radiography is the gold standard [6,7,8,9]. However, due to exposure to radiation, costs, and limited availability, alternative methods have been introduced; their accuracy compared to that of radiography ranges from poor to excellent (Table 1).

To lessen the radiation dose or avoid radiation, short knee radiographs [10,11,12,13], magnetic resonance imaging [14], and motion-capture systems [9,15,16,17,22] are used, although they require specialized equipment and can further increase treatment costs. Other noninvasive methods for FLLA assessment include artificial intelligence-based posture-estimation software [21], digital photography [7,18,19,23,24], goniometry [11,12,13,20,25], and the use of calipers [11,12] or inclinometers [12,26]. Methods based on physical examination are simple and commonly used in screening tests, determining reference values, or follow-up of patients [15,25,27,28,29]. However, clinical methods are highly subjective, especially with regard to the femur axis.

On radiographs, the proximal femoral axis point is set at the center of the femoral head, which is not available in clinical measurements. The highest correspondence with radiographic results was found for digital photography (Table 1). On pelvis-to-floor photographs, the proximal femoral axis point has been set at the greater trochanter [7], at the level of the thigh’s widest circumference [19], and at the estimated center of the hip [18]. However, the anterior superior iliac spine is a common and easily palpable reference point for the femoral axis [26]. Nevertheless, there has been no study on the reliability and accuracy of photography-based FLLA assessment using the anterior superior iliac spine to determine the femoral axis.

Therefore, the purpose of this study was to introduce a new noninvasive photometric method for FLLA assessment and establish its reliability and accuracy. In this method, the anterior superior iliac spine serves as a proximal femoral axis point.

## 2. Materials and Methods

### 2.1. Participants

A total of 67 Caucasian subjects participated in this observational cross-sectional study. Seventeen subjects (6 males and 11 females; average age: 23.1 years, range: 11–47 years) who had been referred for full-length lower limb radiography due to orthopedic disorders (lower limb discrepancy or FLLA deformity) were included to assess the accuracy of the photometric method compared to that of radiography. Fifty healthy subjects (25 males and 25 females; 20.9 years on average, range: 17–24 years) were included to assess the reliability of the photometric method.

All subjects had a normal body mass index (>18.5 kg/m^2^ and <24.99 kg/m^2^), and none had a history of major trauma, i.e., trauma that precludes normal physical activity for more than 1 month. Each subject, including each of the caregivers of minor subjects, was familiarized with the scope of the research and signed the Consent to Participate declaration form before the commencement of the study. The study was performed in accordance with the ethical standards set forth in the 1964 Declaration of Helsinki. All procedures used in this research were approved by the Bioethical Committee at the Poznan University of Medical Sciences (Poland) (decision no. 770/13).

### 2.2. Procedure for Taking Photographs and Radiographs of Subjects with Orthopedic Disorders

In 17 subjects, one pelvis-to-floor lower limb digital photograph and one weight-bearing full-length lower limb radiograph were taken in the standardized position. The standardized position involved the subject standing straight with the lower limbs rotated such that the axis of knee flexion was aligned with the sagittal plane [30]. Radiopaque markers were placed on the skin with double-sided adhesive tape over the anterior superior iliac spines, the distal border of the medial and lateral femoral condyles, and the medial and lateral malleoli, bilaterally. In one subject, only the right lower limb could be assessed due to the presence of an external fixator that had been mounted on the left femur. Radiographs and photographs were taken in immediate succession with the subject standing in the same place in the standardized position. One examiner (AF) prepared the subjects and took digital photographs. Photographs were taken with a Canon PowerShot G11 digital camera (Canon Inc., Tokyo, Japan) attached to a tripod and centered at the subject’s knees. Subjects stood at a distance from the camera that allowed for full-length pictures to be taken of the lower limbs.

### 2.3. Procedure for Taking Photographs of Healthy Subjects

Two pelvis-to-floor lower limb digital photographs were taken in 50 healthy subjects. The anterior superior iliac spines and distal border of the medial and lateral femoral condyles were bilaterally marked with black liner. The first picture was taken with the subject in a standardized position. Next, the marks on the skin were removed by washing. After an hour, the procedure was repeated: the markings were placed on the skin and the second picture was taken. One examiner (AF) prepared the subjects and took digital photographs. Photographs were taken with a Canon EOS 600D digital camera (Canon Inc., Tokyo, Japan) attached to a tripod at a distance from the subjects that allowed for full-length pictures to be taken of the lower limbs, centered at their knees.

### 2.4. Radiographs Analysis

**Figure 1:**
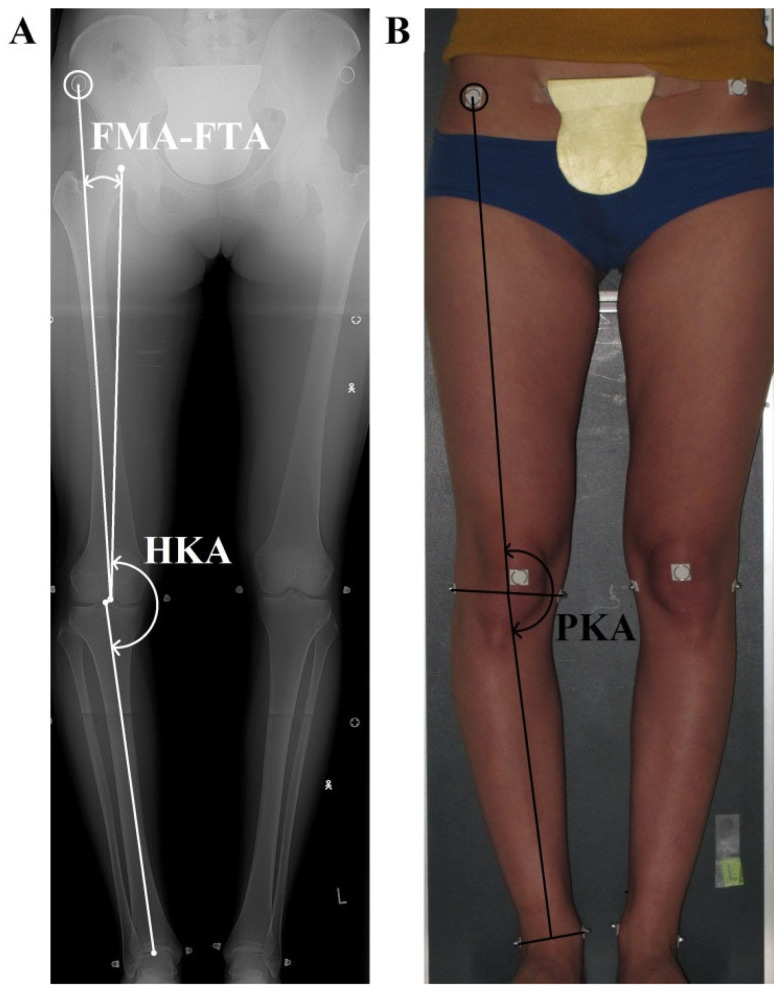
The measured angles. Both pictures were taken of the same patient at the same time, one after the other. The patient stood in the standardized position. (**A**) Radiograph: hip–knee–ankle angle (the HKA, formed by femoral and tibial mechanical axes, with values < 180° indicating varus); the FMA-FTA angle formed by the femoral mechanical axis and the radiographic femoral topographic axis; (**B**) photograph: pelvis–knee–ankle angle (PKA, formed by the photographic femoral and tibial topographic axes).

All pictures were processed in AutoCAD 2017 (Autodesk, San Francisco, CA, USA). On the digital radiographs, the following axes and angles were drawn according to Cooke et al. (2007) [31] (Figure 1A): (1) the femoral mechanical axis (FMA) connecting the center of the femoral head with the center of the femoral notch; (2) the radiographic femoral topographic axis (rFTA) connecting the anterior superior iliac spine with the center of the femoral notch; (3) the tibial mechanical axis (TMA) connecting the center of the tibial intercondylar eminence with the center of the articular surface of the ankle joint; and (4) the hip–knee–ankle angle (HKA), calculated as the medial angle between the FMA and TMA axes. Values below 180° denote varus alignment. Additionally, (5), the FMA–FTA angle was calculated as the angle between the FMA and rFTA axes. The measure of the FLLA on radiographs was the HKA angle value.

### 2.5. Photographs Analysis

All pictures were processed in AutoCAD 2017(Autodesk, San Francisco, CA, USA). On the digital photographs, the following axes and angles were drawn (Figure 1B): (1) the photographic femoral topographic axis (pFTA), which runs from the anterior superior iliac spine to the midpoint of the line connecting the lateral and medial knee joint contour on the level of the distal border of medial and lateral femoral condyles; (2) the tibial topographic axis (TTA), which runs from the distal point of pFTA to the midpoint of the line connecting the most prominent aspects of both malleoli; and (3) the pelvis–knee–ankle angle (PKA), which is calculated as the medial angle between the pFTA and TTA axes. Values below 180° denote varus alignment. The measure of the FLLA on photographs was the PKA angle value.

### 2.6. Accuracy Analysis

To determine the accuracy of the photometric method, the HKA angle on 17 full-length lower limb radiographs and the PKA angle on 17 pelvis-to-floor photographs taken of 17 subjects with orthopedic disorders were measured bilaterally by one rater (AF). In one subject, only the right lower limb could be assessed due to the presence of an external fixator that had been mounted on the left femur. Photographs and radiographs were assessed once by one rater (AF). Intrarater reliability for this rater was calculated using the intraclass correlation coefficient (ICC), yielding ICC(2,k) = 0.999.

### 2.7. Reliability Analysis

To determine the reliability of the photometric method, the PKA angle on 100 photographs taken from 50 healthy participants was measured bilaterally twice by three raters (AF, PK, JSz), with at least a two-week break between the first and second assessments. The raters were blinded to their prior results and to each other’s results. Intrarater reliability was assessed for each rater individually. The interrater reliability was determined for all raters. The test–retest reliability between the first and second photographs was assessed for all raters.

### 2.8. Statistical Analysis

The data were analyzed using STATISTICA 13.0 (StatSoft, Inc., Tulsa, OK, USA). The ICC estimates were calculated using the psych package (version 2.3.12, William Revelle, 2023) [22]. Means and standard deviations of the variables of interest were calculated. The normal distribution of the collected data was confirmed using the Shapiro–Wilk test. Pearson’s correlation coefficient and Bland–Altman analysis were used to examine the relationship and accuracy between the HKA and PKA angles. Simple linear regression was used to develop a regression equation with HKA as the dependent variable and PKA as the independent variable.

The reliability of the PKA angle measurement was evaluated with the ICC and Bland–Altman analysis. Intrarater, interrater, and test–retest reliability coefficients were calculated as ICC estimates, and their 95% confident intervals were calculated based on a mean-rating (k = 2, k = 3 and k = 2 respectively), absolute-agreement, two-way random effects model (ICC(2,k)). Intrarater reliability coefficients between the first and second PKA measurements on the first photograph were calculated for each rater independently with ICC(2,k) and Bland–Altman analysis. Interrater reliability coefficients were calculated for the first PKA measurement on first photograph for all raters or each pair of raters with ICC(2,k) and Bland–Altman analysis, respectively. Test–retest reliability coefficients between the first PKA measurement on the first and second photograph were calculated for all raters with ICC(2,k) and Bland–Altman analysis. ICC estimates were interpreted according to Koo and Li (2016), i.e., <0.5, poor; 0.5–0.75, moderate; 0.75–0.9, good; and >0.9, excellent [32]. Statistical significance was defined at *p* < 0.05.

## 3. Results

### 3.1. The Accuracy of the Photometric Method

In the group of 17 subjects with orthopedic disorders, the mean HKA angle was 178.8° ± 5.2° (range: 168.0–189.6°); the mean FMA–FTA angle was 3.1° ± 1.0° (range: 1.3–4.8°); and the mean PKA angle was 182.6° ± 4.8° (range: 173.1–192.4°).

**Figure 2:**
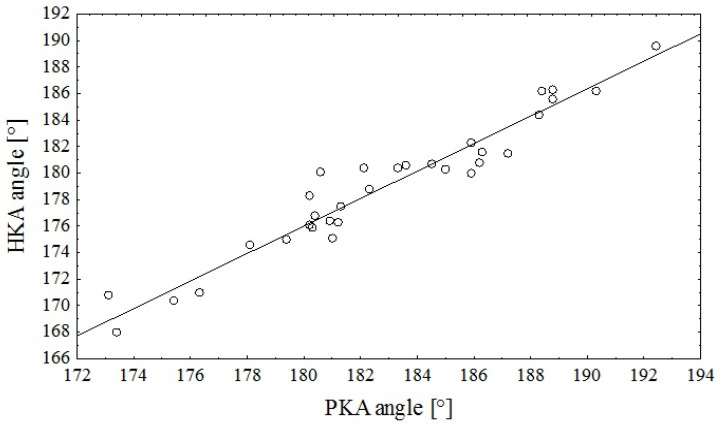
Scatterplot of the relationship between the PKA angle assessed on digital photographs and the HKA angle assessed on digital radiographs (r = 0.97, *p* < 0.0001).

The HKA angle was on average 3.9° ± 1.3° more varus than the PKA angle. Figure 2 shows a strong linear relationship (r *=* 0.97, *p* < 0.0001) between the HKA and PKA angles. In the regression model, the PKA angle alone explained 94% of the variance in the HKA angle (R2 *=* 0.936, *p* < 0.0001). The regression equation was as follows: HKA *=* −10.54 + 1.0364∙PKA. The standard error of the estimate was 1.2427.

The Bland–Altman plot showed a mean bias of −3.891 ± 1.322 between the PKA and HKA angles, and the limits of agreement were −1.300 and −6.482.

### 3.2. The Reliability of the Photometric Method

In the group of 50 healthy subjects, the mean PKA angle was 182.6° ± 2.5° (range 177.3° to 188.0°). The results are presented for the right lower limb. The reliability results are presented in Table 2.

## 4. Discussion

The most important finding in this study was that the photometric method using the anterior superior iliac spine to determine the femoral axis is promising as a reliable and accurate noninvasive tool for assessing FLLA. The advantage of the presented photometric method is the use of the easily palpable anterior superior iliac spine as the proximal femoral axis point. A high correlation between the HKA and PKA angles was found, with the former being approximately 4° more varus than the latter. The prospective design of our study was particularly useful, since it carries a lower risk of confounding and bias. Moreover, the use of the standardized protocol put forth by Cook and Sled (2009) [30] should make our results reliable and reproducible.

Alternative methods to radiography for FLLA assessment are used in screening tests, determining reference values, or follow-up of patients. They can be used in a physiotherapy clinic where there is no access to radiography. However, when making surgical treatment decisions or measuring the outcomes of such operations, radiographs would still be necessary to assess the exact degree of the deformity. Photometry and other non-invasive methods are based on manual landmark identification, which carries a risk of variability. The potential sources of error are differences in body shape, a thick layer of adipose tissue covering bony landmarks in overweight or obese patients, and tight muscle tissue in athletes. The more tissue is present between the bony landmarks and the skin, the more variable the results. Gibson et al. (2010) proposed the umbilical method for assessing FLLA in obese populations; their results were significantly correlated with those from radiography (r = 0.75, *p* < 0.001) [20]. Experience in landmark identification and marking are crucial to obtaining the most reliable measurement. Therefore, even if the results of alternative methods are highly correlated with the results of radiography, the alternative methods are more susceptible to error. 

In the physical examination of FLLA, difficulty arises in regard to defining the proximal femoral axis point, which is the hip-joint center in radiography [6,8,18]. In different studies, various points were used as proximal femoral axis points for FLLA assessment: the center of the thigh [12,13], the umbilicus [20], the midpoint at the level of the thigh’s widest circumference [19], the greater trochanter [7], and the estimated point of the hip center [18,21,23,24,25]. In the photometric method, the anterior superior iliac spine serves as a proximal femoral axis point. This bony landmark is easily palpable. Moreover, FTA defined from the anterior superior iliac spine maintains a consistent relationship with FMA in radiographs. This constant relationship was also found by Wu (2017), who used radiographs to determine the relationship between the femoral mechanical axis and the clinical anatomic axis [26]. The proximal point for the former was set at the hip-joint center, and that for the latter was set at the anterior superior iliac spine; the measurements were taken using a common distal point, the center of the tibial articular surface. The angle between the axes was found to be 4.8° ± 0.2° [26], which is on average 1.7° greater than the FMA–FTA angle obtained in our study. Nonetheless, the difference between the radiographic and photographic angles is ultimately of use in practice. Wu (2017) did not compare radiography against any noninvasive methods [26]. Until now, the reliability and accuracy of methods using the anterior superior iliac spine as the proximal femoral axis reference point have been discussed only by Navali et al. (2012), who used a goniometer to assess FLLA [11]. They found a good correlation (r = 0.674, *p* < 0.0001) between the results of their method and those of radiography [11]. However, they did not present the relationship between the femoral mechanical axis and the femoral axis determined with the anterior superior iliac spine on radiographs [11].

In turn, the definitions of the centers of the knee and ankle joints in physical examination are more consistent across different research works. The center of the knee is usually the midpoint of a line at the level of or near the joint space [18,19,20,23,24,25] or the center of the patella [11,12,13]. The center of the ankle is the midpoint of a line at the level of the malleoli [11,12,13,18,19,20,23,24,25]. For the photometric method, an approach similar to that used in the research mentioned above was adopted. Specifically, the center of the knee joint was defined at the level of the distal border of the femoral condyles, since they are easily palpable. Finally, the center of the ankle joint was defined at the level of the malleoli, which are visible in photographs.

The patient position for FLLA assessment is not consistent across studies; however, it is crucial in alignment measurement [33]. In methods based on digital photography, it has been recommended that the toes be facing forward [7,19,23,24] or that a self-selected stance be adopted [23]. According to Cook and Sled (2009), the plane of knee flexion should be aligned with the sagittal plane to avoid lower limb malpositioning in radiographic measurement [30]. On the other hand, utilizing other distal reference points, such as the patella or tibial tubercle, or having the subjects stand with a fixed rotation of the feet increases the risk of lower limb malrotation [30]. In methods based on digital photography, proper knee position is as important as it is in radiographic measurements if accurate results are to be obtained for the determination of the femoral and tibial axes. Therefore, for the photometric method, the recommendations of Cook and Sled (2009) regarding lower limb positioning were followed [30]. The same approach was adopted by Sheehy et al. (2015) [18].

In practice, the proper positioning of the patient may be time-consuming. However, according to Sun et al. (2021), lower limb rotation contributes to measurement error in FLLA assessment, especially in patients with severe deformities [33]. Nevertheless, the proper positioning of the patient is as important in photography-based methods as it is in radiography. It would be helpful to have a separate space in a clinic for photometric assessment, where a camera could be kept on a tripod at a constant distance from the space for the patient. 

The PKA or HKA angles serve as static measures for lower limb alignment. However, it should be emphasized that in dynamics, e.g., during gait, this alignment changes due to differences in the forces and moments acting on lower limb joints. Böhm et al. (2015) described two cases in which, despite the knee-alignment deformity, the knee moments during gait were normal and became more pathological after surgical treatment [15]. They explained this phenomenon as arising from tibial transverse plane abnormality and advised caution with regard to surgical treatment of knee misalignment when knee mechanics is normal [15]. In their study, Stief et al. (2014) point out that in FLLA deformity, the transverse plane mechanics play a crucial role in load distribution in the knee joint during gait [9]. The study by Donati et al. (2024) highlights the importance of considering interdependence when assessing patients [34]. In their review, they focused on the influence of pelvic position on hip biomechanics, indicating that, e.g., femoral anteversion influences hip internal rotation during gait [34]. Therefore, in treatment planning for FLLA deformities, more measurements, such as pelvic position, assessments of hip rotation or tibial torsion, and, if possible, gait biomechanics analysis, should be considered.

There are several limitations of this study that must be acknowledged, and some areas for further research must be highlighted. First of all, the study sample used for accuracy analysis is relatively small. If the photometric method is to be used in clinical practice, adequate validation against radiography in a sufficiently large and heterogenous study group is necessary. The limited size of this study group did not allow for stratification of results by age, sex, weight, or type and severity of deformity. Therefore, it is not yet known yet how accurate photometry is for deformities of different degrees of severity. What is more, landmark identification in overweight and obese patients is a potential source of error in noninvasive FLLA measurement; therefore, in the next stage, patients with different weights should be examined. In further studies on the accuracy of photometry, subgroup analysis is needed to improve the clinical applicability of this approach. In this study, males and females were assessed together. In a comparison of female and male subjects, Wu (2017) did not find differences in the angle formed by the femoral mechanical axis and the femoral clinical anatomic axis that runs from the anterior superior iliac spine to the center of the knee [26]. Nevertheless, future research on a larger study sample should consider sex when assessing the accuracy of the photometric method. Additionally, radiographs and photographs of patients with lower limb discrepancy or FLLA deformity were obtained only once. It would be valuable to take both pictures on consecutive follow-up visits to assess the reliability and accuracy of the photometric method over time, e.g., pre- and post-surgery. This would allow for the assessment of the method’s clinical applicability and stability over time. Finally, future research should also provide a normative database of healthy subjects in different age ranges.

## 5. Conclusions

In summary, the photometric method is promising as a reliable and accurate noninvasive tool for assessing FLLA. The results of the photometric method are as highly correlated to those of radiography as are those of other methods based on digital photography [7,18,19]. However, the accuracy of this method across different study groups (including subjects who vary in age, sex, weight, and type of deformity) has yet to be confirmed in a larger cohort. The advantage of the presented photometric method is the use of the easily palpable anterior superior iliac spine as the proximal femoral axis point. Considering the relatively small sample size, a study of a larger group is needed to confirm these findings before the photometric method can be recommended for clinical use.

## Figures and Tables

**Table 1:** Correlation coefficients between frontal plane lower limb alignment determined with the gold standard—radiography—and alternative less-invasive or noninvasive methods.

Method	Reference	n *	r *, *p* Value
Short knee radiographs	[10]	n = 200	r = 0.888, *p* < 0.001 (20 cm)
			r = 0.161, *p* = 0.109 (10 cm)
	[11]	n = 100	r = 0.932, *p* < 0.0001 (10 cm)
	[12]	n = 40	r = 0.88, *p* < 0.001 (10 cm)
	[13]	n = 114	r = 0.75, *p* < 0.0001 (10 cm)
Magnetic resonance imaging	[14]	n = 30	r = 0.95, *p* < 0.05
Motion-capture systems	[15]	n = 15	r = 0.62, *p* = 0.012 (1st session)
			r = 0.78, *p* < 0.001 (2nd session)
	[9]	n = 18	r = 0.834, *p* < 0.001
	[16]	n = 20	r = 0.934, *p* < 0.001
	[17]	n = 124	r = 0.738, *p* = 0.001
Digital photography	[7]	n = 39	r = 0.94, *p* < 0.001
	[18]	n = 50	r = 0.92, *p* < 0.0001
	[19]	n = 20	r = 0.98, *p* < 0.001
Goniometer	[11]	n = 100	r = 0.674, *p* < 0.0001
	[20]	n = 55	r = 0.74, *p* < 0.001
	[12]	n = 26	r = 0.32, *p* = 0.12
	[13]	n = 114	r = 0.70, *p* < 0.0001
Caliper	[11]	n = 100	r = 0.899, *p* < 0.0001
	[12]	n = 40	r = 0.76, *p* < 0.001
Inclinometer	[16]	n = 20	r = 0.831, *p* < 0.001
	[12]	n = 40	r = 0.80, *p* < 0.001
Artificial intelligence-based posture-estimation software	[21]	n = 36	r = 0.754, *p* < 0.001

* n—number of examined lower limbs, r—correlation coefficient.

**Table 2:** ICC estimates for intrarater and interrater reliability, as well as test–retest reliability, of the PKA angle (right lower limb) measurement.

Reliability	ICC(2,k) (95% Confident Interval)	Bland–Altman Bias (Limits of Agreement) [°]
**Intrarater**		
Rater 1	0.999 (0.999, 0.999)	−0.03 (−0.24, 0.19)
Rater 2	0.993 (0.988, 0.996)	0.08 (−0.67, 0.82)
Rater 3	0.972 (0.950, 0.984)	−0.07 (−1.65, 1.51)
**Interrater**		
Raters 1 & 2 & 3	0.991 (0.986, 0.995)	-
Rater 1 & 2	-	0.01 (−0.33, 0.35)
Rater 1 & 3	-	0.1 (−1.12, 1.34)
Rater 2 & 3	-	0.1 (−1.19, 1.39)
**Test–retest**		
Photographs 1 & 2	0.980 (0.973, 0.986)	0.04 (−1.28, 1.35)

## Data Availability

The datasets used and/or analysed during the current study are available from the corresponding author on reasonable request.

## Abbreviations

The following abbreviations are used in this manuscript:

FLLA	frontal lower limb alignment
HKA	hip–knee–ankle
PKA	pelvis–knee–ankle
FMA	femoral mechanical axis
rFTA	radiographic femoral topographic axis
TMA	tibial mechanical axis
pFTA	photographic femoral topographic axis
TTA	tibial topographic axis
ICC	intraclass correlation coefficient
